# Digital Health Tools Embedded in a Cancer Genetics Clinic: Observational Study

**DOI:** 10.2196/74375

**Published:** 2026-02-02

**Authors:** Sujay Nagaraj, Ron Rabinowicz, Sarah Goodday, Ledia Brunga, Chana Korenblum, Anita Villani, Raymond Kim, Emma Karlin, Robert William Greer, Hadrian Balaci, Meis Omran, Anna Goldenberg, David Malkin, Stephen Friend

**Affiliations:** 1 Department of Computer Science University of Toronto Toronto, ON Canada; 2 The Hospital for Sick Children Toronto, ON Canada; 3 Schneider Children's Medical Center Petah Tikvah Israel; 4 4YouandMe Seattle, WA United States; 5 Department of Psychiatry University of Oxford Oxford United Kingdom; 6 Department of Paediatrics University of Toronto Toronto, ON Canada; 7 Department of Psychiatry University of Toronto Toronto, ON Canada; 8 Princess Margaret Cancer Centre Toronto, ON Canada; 9 Department of Medicine University of Toronto Toronto, ON Canada; 10 Department of Oncology-Pathology Karolinska Institutet and Karolinska University Hospital Stockholm Sweden; 11 Genetics and Genome Biology Program The Hospital for Sick Children Toronto, ON Canada; 12 Department of Laboratory Medicine and Pathobiology University of Toronto Toronto, ON Canada; 13 Department of Molecular Genetics University of Toronto Toronto, ON Canada; 14 Department of Medical Biophysics University of Toronto Toronto, ON Canada

**Keywords:** digital health, cancer, wearables, pediatrics, engagement

## Abstract

**Background:**

Digital Health Tools (DHTs), including wearables and mobile apps, offer promising avenues for personalized care and real-time monitoring, but user engagement and clinical utility—especially in pediatric populations—remain unclear. Li-Fraumeni syndrome (LFS) is a genetic mutation in the *TP53* tumor suppressor gene, predisposing individuals to cancer, requiring lifelong surveillance and associated psychological stress.

**Objective:**

We evaluated engagement with DHTs in a cancer genetics clinic for families affected by LFS and explored their utility for patients and clinicians. Our goal was to identify insights that could inform future integration of DHTs in chronic disease populations and contribute to research.

**Methods:**

We conducted an observational study (January-December 2022) involving patients with LFS and family members aged 5 years and older. Participants received an Empatica EmbracePlus smartwatch and a suite of self-report surveys assessing psychosocial well-being at varying frequencies (ie, daily, weekly, etc). We used survival analysis to characterize engagement over time across age, *TP53* status, and previous cancer history. Generalized additive models were used to explore physiological patterns relative to cancer surveillance events. Semistructured interviews provided qualitative insight into user experiences and preferences.

**Results:**

We enrolled 9 children and 36 adults. Adults wore their smartwatches more often than children (mean 81%, SD 19% vs mean 56%, SD 26%; *t*_10_*_._*_1_=2*.*72; *P*=*.*02) and were engaged in the study for a longer duration (median retention 153, IQR 119-179; 95% CI 133-177 vs median 77, IQR 36-151; 95% CI 17-171 days; log-rank *χ*^2^_1_=4*.*4; *P*=*.*04). Daily wear time was similar between the 2 groups (mean 17.6, SD 3.1 hours vs mean 15.7, SD 2.9 hours; *t*_13_*_._*_2_=1*.*70; *P*=*.*11). There were no differences in survey engagement between adults and children, nor were there differences in engagement across *TP53* status or previous cancer history. Children reported greater psychosocial burden, with more depressive symptoms (PHQ-9 [Patient Health Questionnaire-9] score mean 10.0, SD 5.2 vs mean 4.2, SD 4.4; *t*_7_*_._*_8_=2*.*8; *P*=*.*03), worse sleep (PROMIS SRI [patient-reported outcomes measurement information system sleep-related impairment] score mean 22.7, SD 5.9 vs mean 16.5, SD 5.5; *t*_8_*_._*_1_=*−*2*.*58*; P*=*.*03), and increased frequency of stress (mean 36.3%, SD 19.9% vs mean 14.3%, SD 19.2%; *t*_8_*_._*_3_=*−*2*.*7; *P*=*.*03) than adults. A suicide alert system was triggered in 5 participants (11%) and prompted timely clinical intervention. Generalized additive model analysis showed individualized yet consistent physiological patterns of stress associated with cancer surveillance. Qualitative feedback from participants identified perceived value in stress awareness, but highlighted challenges with device comfort, functionality, and personalization.

**Conclusions:**

DHTs are feasible and can capture clinically meaningful psychological and physiological data in high-risk pediatric and family populations with LFS. They enable timely detection of distress and facilitate targeted interventions. Our findings can inform best practices for patient-centered DHT integration into clinical care, with relevance to pediatric oncology and broader digital health contexts.

## Introduction

Digital Health Tools (DHTs)—wearable devices, smartphone apps, and remote monitoring systems—are being integrated into clinical care [1,2]. DHTs promise enhanced physiological monitoring in settings outside traditional health care touchpoints (eg, home and work) [3,4], improved treatment and study engagement through digital notifications, and longitudinal collection of patient-reported outcomes [5,6]. DHTs are often paired with study apps for activity annotation and event flagging, enabling feedback and longitudinal outcome measures [7,8]. One specific use case that offers promise is the use of DHTs to monitor chronic disease progression [9-12]. Wearable sensors can capture physiological states outside clinical settings, encompassing not only disease biology but also the lived experiences (eg, psychosocial consequences) and the iatrogenic impacts of interventions, through serial patient-reported outcomes via app-based measures.

In our study, we focus on the application of DHTs for individuals and families affected by Li-Fraumeni Syndrome (LFS; MIM 151623) at a major Canadian hospital network. LFS is a genetic disorder caused by mutations in the *TP53* tumor suppressor gene, significantly increasing the risk of developing cancer from an early age [13,14]. Individuals with LFS face a lifetime cancer risk of approximately 40% by 20 years of age and over 90% by 70 years of age, requiring ongoing cancer surveillance using modalities such as magnetic resonance imaging (MRI) [15]. Survivors of a primary cancer have an 83-fold risk of developing subsequent malignancies. Clinical surveillance protocols [16] have significantly reduced mortality and treatment-related morbidity. However, the continuous cancer threat and intensive surveillance impose considerable psychological and emotional burdens on affected individuals and their families. Notably, ‘scanxiety’ is a period of emotional distress around cancer surveillance [17]. This impacts entire families—not only through the genetic nature of LFS but also the threat of future cancer diagnoses in loved ones and the shared experience of repeat surveillance.

DHTs show significant potential in providing clinicians with deeper insights into the lived experiences of patients with LFS and the iatrogenic impacts of cancer surveillance protocols. However, the extent to which this potential can be realized remains a blind spot in research. Recently, the World Health Organization outlined a classification of digital health interventions [18]—we propose addressing our study objectives on 3 fronts in accordance with the taxonomy described in their report: engagement with DHTs, benefits for health care providers, and benefits for patients. These domains are described below:

1. Engagement. Do patients want to use DHTs, and will they? This section addresses engagement with DHTs among families with LFS. We study the willingness of patients to use DHTs and the factors influencing their engagement.
2. Clinician benefits. How can DHTs benefit clinicians? This section explores the potential advantages DHTs offer to health care providers.
3. Patient benefits. How can DHTs benefit patients and families? This section explores the positive impacts of DHTs identified by patients and their families.

Our study builds on existing work studying the integration of DHTs into health care [8,10,19,20]. We study the role of DHTs in a unique patient population (children and adults with cancer predisposition), with an emphasis on benefits to multiple stakeholders (ie, clinicians and patients), and we identify unforeseen consequences from such interventions. We intend for this work to inform how DHTs can be integrated into cancer clinics and health care in general.

## Methods

### Study Design

This observational study collected longitudinal data via repeat survey measures and wearable technologies from individuals and families affected by LFS. Participants aged 5 years and older were recruited from the cancer genetics program clinics at The Hospital for Sick Children (SickKids) and the Princess Margaret Cancer Center, with Research Ethics Board (REB) approval from both institutions (REB 1000072240). As outlined in Textbox 1, families of at least one affected member with LFS, including affected and unaffected relatives, were recruited during routine follow-up visits. A clinical fellow (RR) conducted biweekly check-in calls with families to support participation and gather insights on their study experience, disease, and familial burden. These calls provided rich qualitative data to inform future digital health studies for high-risk families. Recruitment for the study was done on a rolling basis from January to June 2022, as families presented to the clinic for their usual appointments. The target follow-up period was 6 months (180 days) for each participant, with a study end date of December 2022.

**Inclusion and exclusion criteria for study recruitment.**

**Inclusion criteria**

- Families in whom either a parent or a child is affected with Li-Fraumeni syndrome (confirmed pathogenic germline variant in TP53).
- A noncarrier family member from a family where either a parent or a sibling is a confirmed carrier of a pathogenic germline variant in TP53.
- Able to comfortably wear the Empatica EmbracePlus band on the wrist or ankle.
- Able to speak, write, and read English (not applicable for younger children where assent was sought).
- Able to provide informed consent or assent (in children).
- Adult participants must have a personal smartphone and be willing to use their phone for the study, including downloading study apps and syncing devices (locked phones provided for participants aged 11 years or older without phones).

**Exclusion criteria**

- An active cancer diagnosis, undergoing active cancer therapy at enrollment, less than 6 months post systemic cancer therapy (except hormonal therapy for breast cancer), or less than 3 months post cancer surgery if no other therapy is recommended.
- Women who are pregnant or planning to become pregnant within 6 months.

### Study Measures and Preprocessing

#### Overview

Study participants were given an EmbracePlus smartwatch built by Empatica Inc. Participants were also instructed to download the MyCap study app developed by REDCap (Research Electronic Data Capture; Vanderbilt University) on their phone for repeat survey administration. The study was designed to capture both passive (smartwatch) and active (survey) information longitudinally. We intentionally chose devices and platforms that are already approved for clinical use and can therefore be scaled easily for future work.

#### Smartwatch

The EmbracePlus smartwatch uses the Empatica Health Monitoring Platform and is certified with 510k clearance (US Food and Drug Administration) and as a class IIa medical device (European Union). It features several sensors:

1. Ventral electrodermal activity (EDA): detects subtle changes in electrical conductance at the surface of the skin.
2. Optical photoplethysmogram: measures heart rate and heart rate variability (HRV).
3. Digital skin temperature: reads peripheral skin temperature.
4. Accelerometer and gyroscope: collect raw acceleration and angular velocity data.

These physiological parameters are sampled at 64 Hz and sent via Bluetooth to a dedicated mobile app. This app automatically syncs all data to the Empatica Cloud and is compatible with iOS and Android smartphones. The smartwatch has previously been validated in clinical and research settings to monitor patient health via detection of physiological changes [21,22].

#### Study App

The MyCap study app administered daily, weekly, biweekly, and monthly surveys. Responses were stored on a secure REDCap instance at SickKids Hospital. Specific, age-appropriate surveys were administered to different age groups. For children aged 5-10 years, parents completed the surveys on behalf of their children. The MyCap app facilitated continuous engagement and data collection, allowing us to gather comprehensive information on participants’ health and well-being. For a full list of measures, descriptions, rationale for selection, and the frequency of administration, please see Tables S1-S3 in Multimedia Appendix 1. Each survey was selected based on several factors: previous validation in the specific age group administered, as well as previous utility in similar study designs. They also contain a set of both subjective and objective measures of stress and well-being.

### Data Management and Deployment

Our dataset comprised 3 sources: the Epic electronic health record, the Empatica Smartwatch, and the study app. Epic provided baseline clinical information, including patient demographics. The smartwatch collected various physiological data streams stored on Amazon S3, with participants able to access their own data via Empatica. Participants did not have access to their completed survey responses.

Upon study completion, data from these sources were transferred to a secure SickKids server with high-performance computing (HPC) capabilities. The HPC environment facilitated the integration, merging, and preprocessing of records, enabling analysis from all 3 data sources. This data management system marked the first use of such a pipeline for DHTs at SickKids hospital, establishing a framework for future clinical applications of DHTs (Figure S1 in Multimedia Appendix 1).

### Analysis

#### Statistical Analyses

We compared overall survey completion and smartwatch use rates between subgroups (ie, adults vs children, *TP53* wild-type [wt] vs mutant [mut], and cancer history vs none) using 2-sample *t* tests with Welch correction. Unlike a 2-tailed t test, the Welch t test does not assume equal variances or balanced group sizes, making it more robust to heteroscedasticity in real-world engagement data. All *P* values are 2-tailed and were rounded to 3 decimal places.

#### Engagement Survival Analysis

Participant engagement was assessed quantitatively via adherence to daily surveys and smartwatch usage, and qualitatively through biweekly check-ins and clinical visits exploring barriers and benefits to participation.

Engagement metrics across digital health studies vary widely [23], so we explicitly outline our metrics here. Overall, survey engagement was quantified as the percentage of daily surveys completed per participant over the follow-up period (specifically, the Daily Stress Measure described in Table S1 in Multimedia Appendix 1). We also calculated rolling weekly averages of survey completion rates to examine how engagement fluctuates over time. Overall, smartwatch engagement was defined as the proportion of days participants wore the smartwatch for at least 6 hours, excluding improper wear artifacts identified by the manufacturer’s algorithm. We also computed rolling weekly averages to represent dynamic engagement trends. This analysis does not differentiate between awake and sleep wear, though we separately analyzed awake versus sleep wear distribution by age group (Table 1).

**Table 1:** Participant characteristics and smartwatch wear metrics by age category.

Measure		Child (*<*18 years; n=9)	Adult (≥18 years; n=36)
LFS^a^ (*TP53* mutation), n (%)		9 (100)	26 (72)
Cancer history, n (%)		4 (44)	10 (29)
Age (years), mean (range)		15.1 (7.9-18)	43.1 (22.8-68.1)
Daily survey completion rate (%), mean (SD)		51 (24)^b^	65 (22)
**Smartwatch** **wear** **(≥6 hours/day)**			
	Smartwatch wear (days), mean (SD)	58 (56)	86 ( 50)
	Days worn (%), mean (SD)	56 (26)	81 (19)
	Total wear time (hours), mean (SD)	15*.*7 ( 2*.*9)	17*.*6 ( 3*.*1)
	Sleep time (hours), mean (SD)	3*.*7 ( 2*.*2)	4*.*4 ( 2*.*1)
	Awake time (hours), mean (SD)	6*.*8 ( 4*.*2)	9*.*1 (4*.*4)
**Smartwatch** **wear** **(≥1 hour/day)**			
	Smartwatch wear (days), mean (SD)	65 ( 58)	93 ( 50)
	Days worn (%), mean (SD)	62 (25)	86 (16)
	Total wear time (hours), mean (SD)	14*.*0 ( 3*.*5)	16*.*5 ( 3*.*5)
	Sleep time (hours), mean (SD)	3*.*3 ( 2*.*1)	4*.*2 ( 2*.*0)
	Awake time (hours), mean (SD)	6*.*1 ( 4*.*0)	8*.*6 ( 4*.*3)

^a^LFS: Li-Fraumeni syndrome.

^b^Daily survey completion rate calculated for children aged 11-17 years only (n=7).

To compare engagement patterns across subgroups (eg, age), we used survival analysis. Measuring smartwatch engagement is challenging due to intermittent usage patterns. Therefore, we adopted the methodology described by Cho et al [20] to define participation end points for survival analyses clearly (see original paper for a detailed description). Briefly, each observed day was classified into 1 of 2 states: active if smartwatch wear exceeded a defined threshold (*≥*6 hours), or nonactive otherwise. Similarly, survey days were classified as active upon completing the Daily Stress Measure and nonactive if incomplete. Initially, all nonactive days were labeled exit; these were reclassified as inactive if followed by subsequent active days, reflecting temporary disengagement. Survival analysis used the first observation as time 0 days and 6 months (time 180 days) as the end date. Participants with events after this interval were considered censored. We modeled smartwatch and survey engagement where the event is defined as the first sustained exit within 180 days. Individuals who did not have an exit event were deemed as censored. Kaplan-Meier curves were created to model the survival functions generated by this analysis, and log-rank tests were used to compare median engagement across demographic subgroups.

#### Temporal Analysis

We conducted temporal (time series) analysis to characterize time-dependent patterns in survey and smartwatch adherence. Both survey and smartwatch adherence data for each engagement were smoothed using a centered 7-day rolling average. For each participant, we then computed the Pearson correlation coefficient between the 2 smoothed time series (ie, smartwatch and survey adherence). To characterize seasonal patterns in individual engagement, we applied a real-valued Fast Fourier Transform (FFT) to each detrended daily series. Specifically, for each participant’s smoothed time series of engagement metrics, we subtracted the sample mean to detrend each time series then computed the FFT to transform data to the frequency domain. We then converted positive frequencies to periods in days (ie, inverse of frequency is period). Dominant periods were identified as the 3 largest spectral magnitudes. This FFT-based periodogram method has been used previously to reveal weekly, monthly, and other cyclic patterns in wearable sensor data [8].

#### Generalized Additive Model

To investigate the phenomenon of “scanxiety”—heightened anxiety and stress before clinical surveillance scans (eg, MRI to detect tumor recurrence or remission)—we used generalized additive models (GAMs) [24]. GAMs were used to model the relationship between the time-varying physiological measures from the smartwatch and proximity to each scan (ie, time-to-event regression). Their flexibility in capturing nonlinear relationships with cubic splines and interpretability made them ideal for this task.

Using the *pygam* library [25], separate GAMs were fit for each surveillance event (eg, participant A’s MRI) with default hyperparameters. High-frequency smartwatch data (minute-level) up to 3 days before each scan provided thousands of data points per event.

Data preprocessing (Figure S2 in Multimedia Appendix 1) included imputation of missing values via multivariate imputation via chained equations (MICE) and feature normalization (log EDA, heart rate, log HRV, respiratory rate, and temperature) to zero mean and unit variance. We note that HRV and EDA were log-transformed to reduce positive skew and stabilize variance, ensuring approximate normality and improving model interpretability. A feature capturing missingness at each time step was included as a proxy for imputed data. This was done to study any possible relationship between missingness and the scan event, as we assume that data are not missing at random. Nonlinearities were modeled using cubic splines, and restricted maximum likelihood estimation was used to fit each GAM. Model performance was assessed using mean squared error from 5-fold cross-validation, and partial dependency plots visualized feature relationships with scan proximity (Table S4 in Multimedia Appendix 1).

Participants included in this analysis were those with at least 2 scans and at least 300 hours of smartwatch wear-time leading up to each scan. The final cohort consisted of 2 children and 7 adults. This was done to study inter- and intraindividual differences in the physiological sensor data within proximity to scan events.

#### Qualitative Experiences

Upon study completion, we interviewed each family to gather feedback on successes and challenges in the study design. We did not conduct a formal qualitative study or thematic analysis. Instead, qualitative insights were collected as part of routine operational monitoring. Telephone conversations were not recorded or transcribed; the study coordinator (author RR) documented them in semistructured free-text fields in REDCap, guided by a priori domains (eg, device usability, barriers to engagement, and emotional response to alerts) while allowing open-ended notes.

These entries were reviewed during biweekly team meetings, where common themes were identified through discussion and used to adapt the monitoring protocol. Some qualitative findings were supported by quantitative data (eg, frequency of PHQ-9 [Patient Health Questionnaire-9] alerts). We acknowledge that the absence of formal qualitative methods, coding, or interrater checks limits reproducibility, but we aimed to offset this with consistent documentation, reflexive team discussion, and integration of participant-reported experiences with engagement metrics.

### Ethical Considerations

This study was reviewed and approved by the REB at SickKids Hospital, Toronto, Ontario, Canada (REB 1000072240), in accordance with the ethical standards outlined in the 1964 Declaration of Helsinki and its later amendments. All participants provided written informed consent before enrollment in the study. The consent process included agreement to the collection of both quantitative and qualitative data, as well as the use of anonymized responses for research publication purposes. No additional consent was required for the analysis presented in this manuscript, as it fell within the scope of the original ethics approval and participant consent. All data used in this study were deidentified before analysis to protect participant privacy and confidentiality. No identifying information was linked to the analysis datasets, and access to the raw data was restricted to authorized members of the research team in compliance with institutional data governance policies. No compensation was provided to participants for their involvement in the study.

## Results

### Recruitment

**Figure 1:**
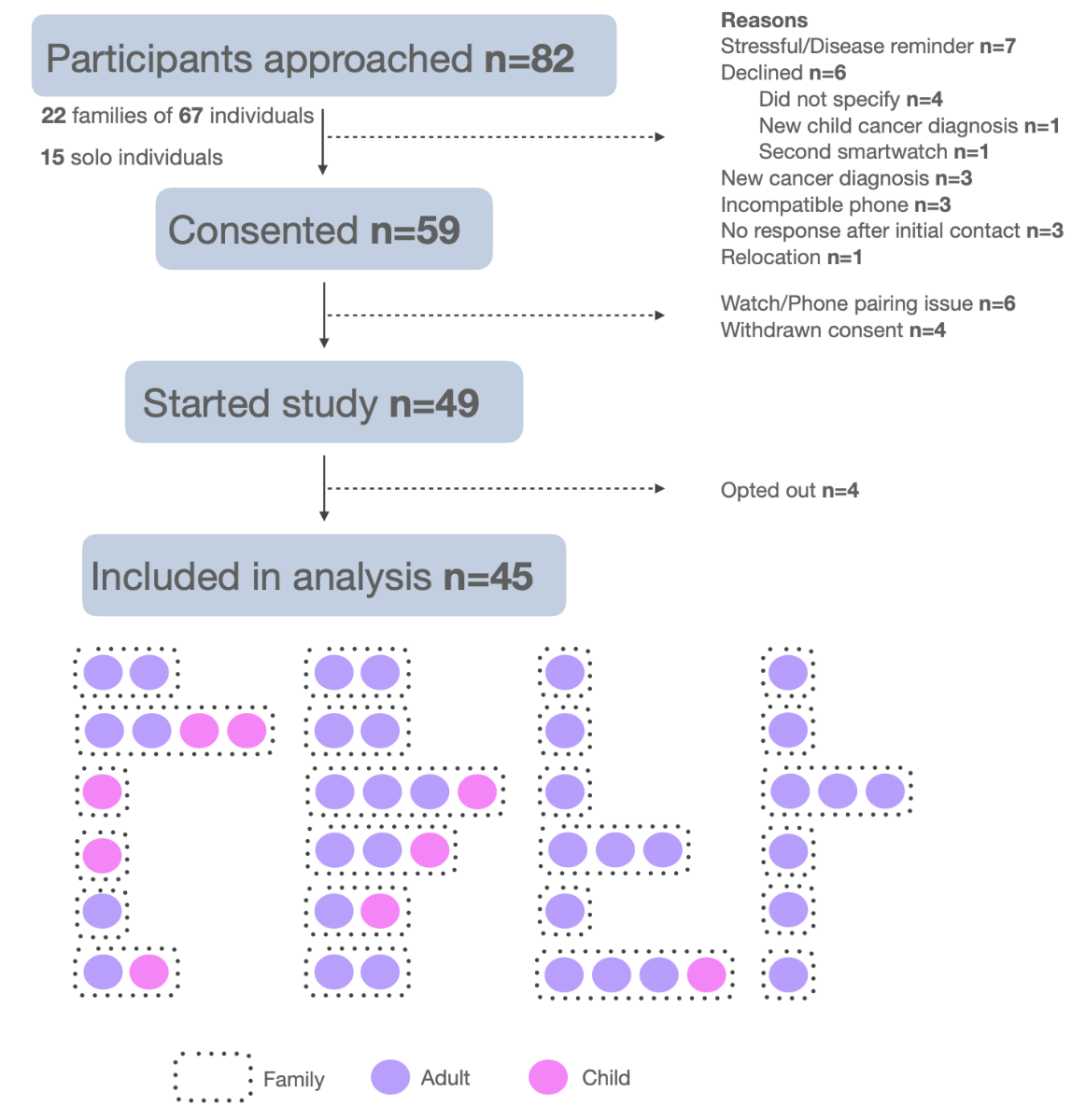
Flowchart detailing participant recruitment and retention in the study. Out of 82 participants approached, 59 consented, 49 started the study, and 45 were included in the final analysis.

A total of 82 individuals were approached for enrollment, including 22 families (67 individuals) and 15 solo individuals. Of these, 59 (72%) individuals provided informed consent. Reasons for refusal included stress concerns related to their disease (n=7), recent cancer diagnosis (n=3), incompatible mobile phone (n=3), nonresponsiveness (n=3), recent cancer diagnosis in a child (n=1), owning a second smartwatch (n=1), relocation (n=1), and unspecified reasons (n=4). Among consenting participants, 49 initiated the study, 6 experienced technical issues pairing the smartwatch, and 4 withdrew consent. Ultimately, 45 participants were included in the final analysis, as 4 did not wear the watch for unspecified reasons. The cohort comprised 12 families (each with at least 2 related participants) and 12 solo participants. Study recruitment and final cohort demographics are detailed in Figure 1.

The cohort was diverse in age, *TP53* status (*TP53* wt: unaffected, *TP53* mut: affected), and other demographics (Table 1). Unfortunately, we were unable to recruit child noncarriers (ie, *TP53* wt). We flag this limitation for readers as differences between carriers and noncarriers may be confounded by adult and child differences.

### Engagement

Long-term engagement in DHT-based studies is not only challenging to ensure, but it is also difficult to measure [20,23]. The difficulty in measurement can be attributed to the multitude of definitions for “engagement” when using repeated measures (ie, how to properly define disengagement). To circumvent these limitations and provide a holistic description of our study’s engagement statistics, we considered both cohort-level and individual-level engagement rates.

**Figure 2:**
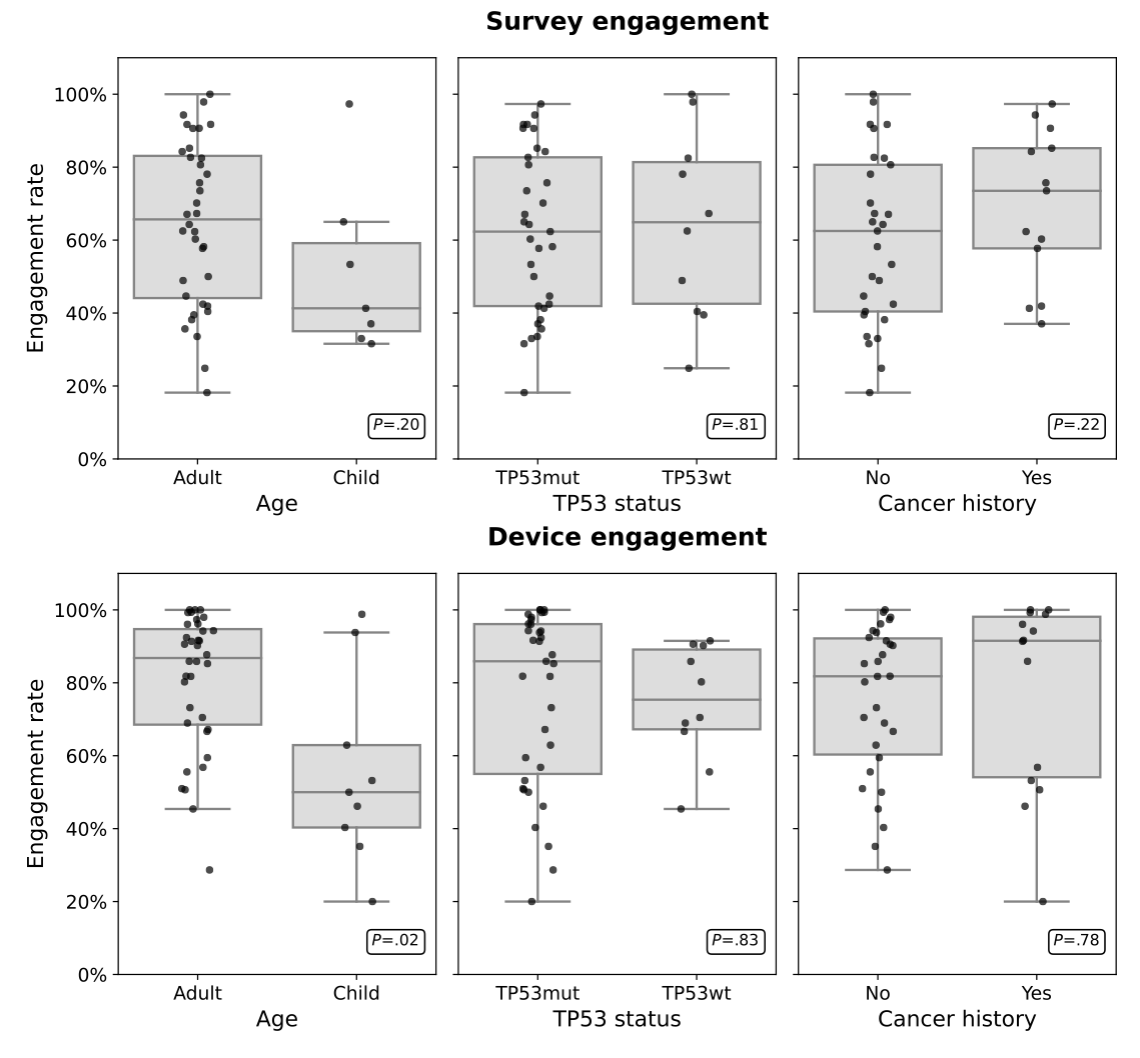
Overall survey and device engagement rates across the study duration, stratified by age group (left), TP53 status (middle), and cancer history (right).

Cohort engagement refers to the overall completion rates of survey engagement and the total wear time of the smartwatch during the follow-up period. As described in the “Methods” section, we define smartwatch engagement as 6 hours or more of proper wear time per day and survey engagement as % of daily survey instances completed during the follow-up period. We can see from Table 1 and Figure 2, all participants demonstrated high engagement for survey completion with no significant differences between adults and children (mean 65%, SD 22% vs mean 51%, SD 24%; *t*_8_*_._*_3_=1*.*39; *P*=*.*20). When comparing across different subgroups, survey engagement did not significantly differ between *TP53* wt and *TP53* mut participants mut (mean 64%, SD 26% vs mean 62%, SD 22%; *t*_13_*_._*_5_=*−*0*.*25; *P*=*.*81) or between those with previous cancer history and those without (mean 69%, SD 21% vs mean 60%, SD 23%; *t*_25_*_._*_8_=*−*1*.*25*; P*=*.*22). Interestingly, adults had better smartwatch engagement than children (mean 81%, SD 19% vs mean 56%, SD 26%; *t*_10_*_._*_1_=2*.*72; *P*=*.*02). Though on days where devices were worn at least 6 hours, wear time was similar between the 2 groups (mean 17.6, SD 3.1 hours vs mean 15.7, SD 2.9 hours; *t*_13_*_._*_2_=1*.*70; *P*=*.*11). Both adults and children wore their devices while sleeping for at least 3 hours per night (mean 4.4, SD 2.1 hours vs mean 3.7, SD 2.2 hours; *t*_11_*_._*_7_=0*.*83; *P*=*.*42). Daytime wear was also at least 6 hours per day on average between the 2 groups (mean 9.1, SD 4.4 hours vs mean 6.8, SD 4.2 hours; *t*_12_*_._*_8_=1*.*44*; P*=*.*17). There were no differences in smartwatch engagement rates across *TP53* wt and *TP53* mut (mean 75%, SD 16% vs mean 76%, SD 24%; *t*_22_*_._*_2_=0*.*22; *P*=*.*83) or between those with previous cancer history and those without (mean 77%, SD 26% vs mean 75%, SD 21%; *t*_21_*_._*_1_=*−*0*.*29; *P*=*.*78). There was also a strong coupling of engagement rates when studying the correlation between smartwatch and survey engagement rates across the study population (Figure S3 in Multimedia Appendix 1).

**Figure 3:**
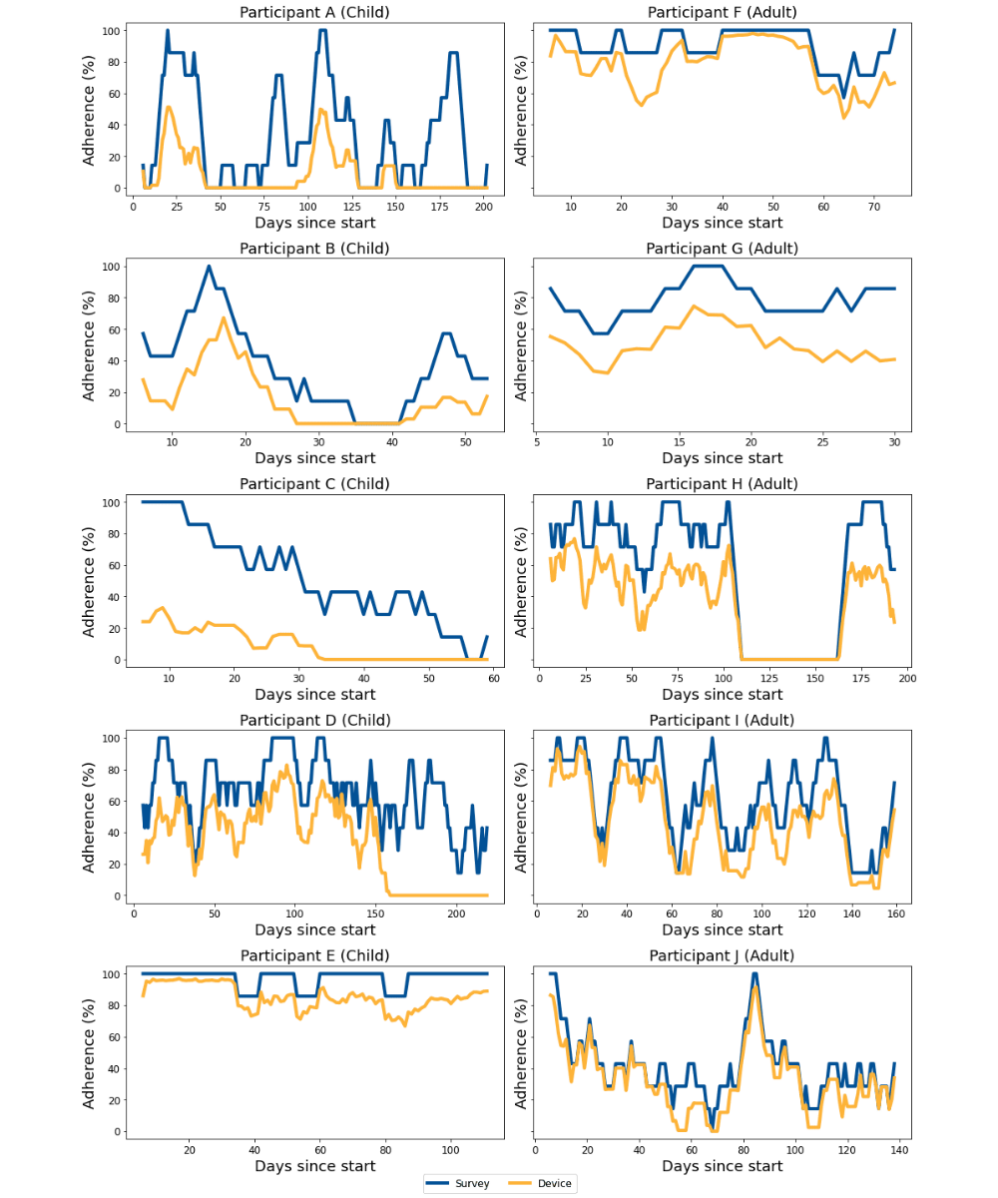
Engagement rates to smartwatch usage (blue) and survey completion (yellow) over time for randomly selected participants, separated by age group (left column: children, right column: adults).

**Figure 4:**
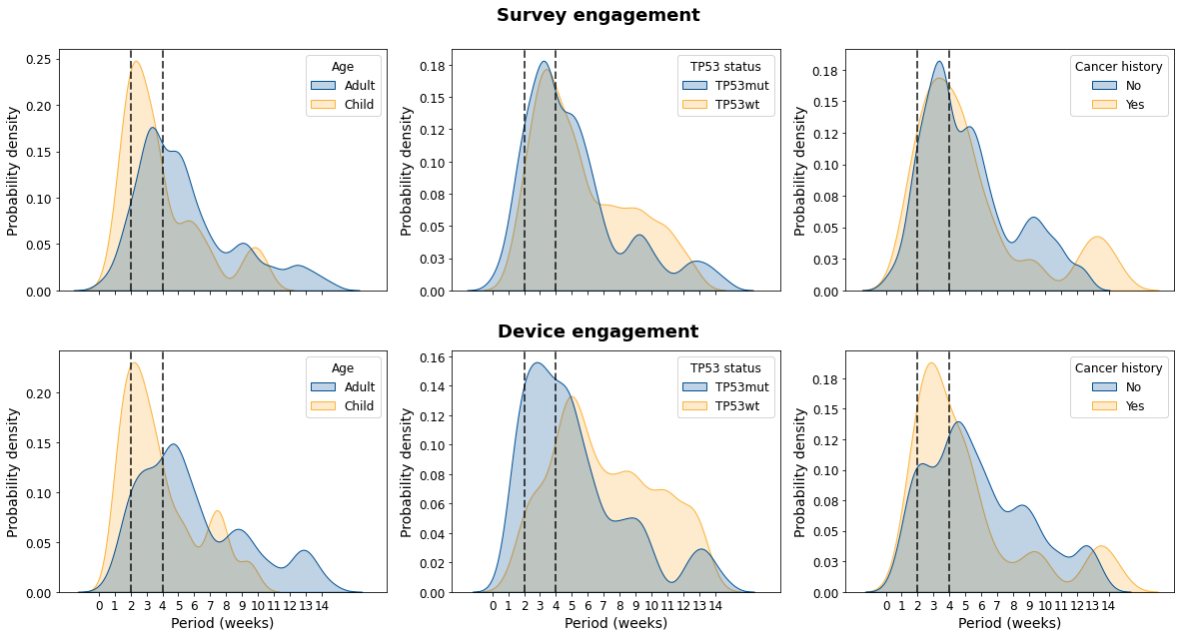
Top 3 peak period distribution (weeks) after Fast Fourier Transform decomposition of survey and smartwatch engagement rates stratified by TP53 status, age, and cancer history.

To study individual-level engagement patterns across time, we plotted engagement over time. Qualitatively, there was substantial inter- and intraindividual variability in engagement alignment, with survey and smartwatch rates fluctuating both between and within individuals (Figure 3). Notably, strong seasonal trends with peaks, troughs, and declining engagement were observed. FFT analyses identified pronounced biweekly and monthly engagement cycles aligning with our biweekly clinical check-ins with participants (Figure 4). This was consistent across all strata (age, *TP53* wt status, and cancer history). The dashed line in Figure 4 annotates 2 to 4 weeks.

**Figure 5:**
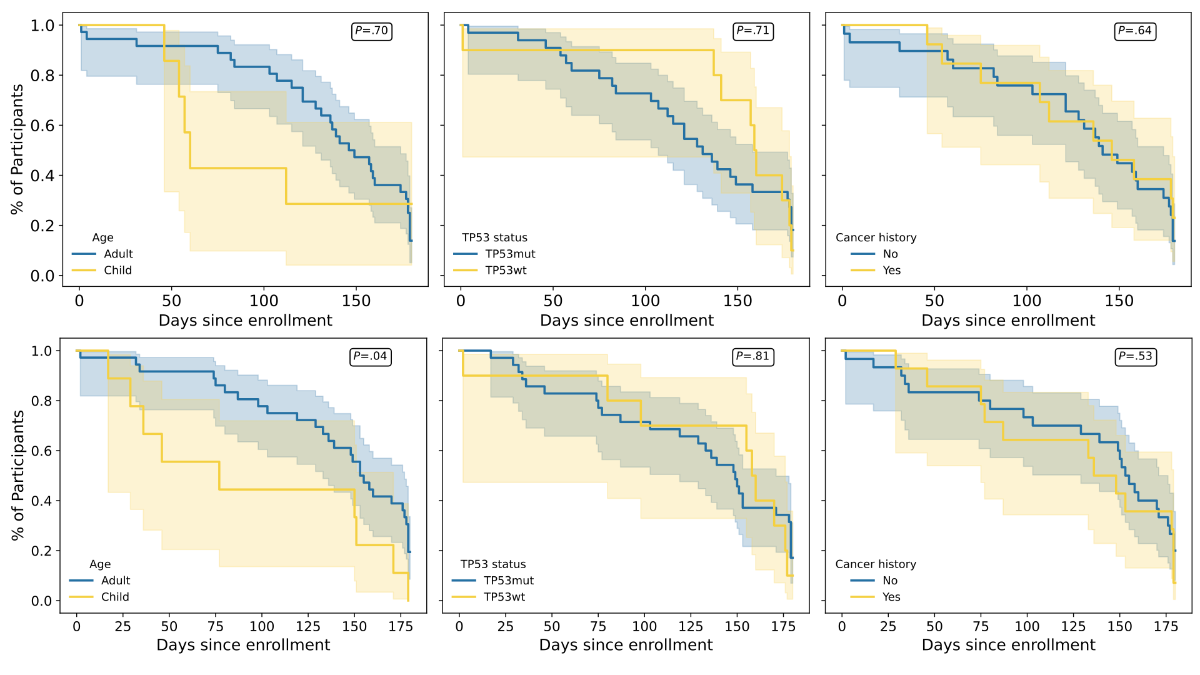
Plots showing the survey retention (top row) and smartwatch retention (bottom row) over time since enrollment, stratified by TP53 status (left column), age group (middle column), and previous cancer history (right column).

Finally, survival analysis uncovered interesting differences in study retention across age groups. Median survey retention was 146 (IQR 121-178; 95% CI 128-174) days for adults versus 60 (IQR 54-NA; 95% CI 46-NA) days for children, with no significant difference between groups (log-rank *χ*^2^_1_=0*.*2; *P*=*.*70). By *TP53* status, median retention was 131 (IQR 84-179 days; 95% CI 107-158) for *TP53* mut participants versus 160 (IQR 141-178 days; 95% CI 1-178) for *TP53* wt participants (log-rank *χ*^2^_1_=0*.*1; *P*=*.*71). Participants with a cancer history had a median retention of 146 (IQR 107-179; 95% CI 7-179) days compared to 141 (IQR 103-178; 95% CI 121-174) days for those without, with no significant difference observed (log-rank *χ*^2^_1_=0*.*2; *P*=*.*64). In Figure 5, the bottom row represents smartwatch engagement, and the top row represents survey engagement as defined in the “Methods” section. Number of participants at t=0: TP53 mut - 32, TP53 wt - 9, adult - 34, child - 7 (9 for smartwatch), cancer History - 27, no cancer history - 13. In this figure, we see that median smartwatch retention was 153 (IQR 119-179; 95% CI 133-177) days for adults versus 77 (IQR 36-151; 95% CI 17-171) days for children. Adults demonstrated significantly greater smartwatch retention compared to children (log-rank *χ*^2^_1_=4*.*4; *P*=*.*04). By *TP53* status, retention was 149 (IQR 77-179; 95% CI 119-171) days for *TP53* mut participants versus 158 (IQR 98-176; 95% CI 2-176) days for wt participants, with no significant difference (log-rank *χ*^2^_1_=0*.*06; *P*=*.*81). Participants with a cancer history had a median retention of 148 days (IQR 77-179; 95% CI 75-179) days compared to 153 (IQR 98-179; 95% CI 129-176) days for those without, with no significant difference (log-rank *χ*^2^_1_=0*.*4; *P*=*.*53).

### Revealing Insights Into Patient Psychosocial State

Patient-reported Outcomes Measurement Information System Sleep-related Impairment (PROMIS SRI), PHQ-9, Perceived Stress Scale-4 (PSS-4), and Generalized Anxiety Disorder-7 (GAD-7) among participants reveal important insights into the psychological and emotional distress experienced by different participants.

**Figure 6:**
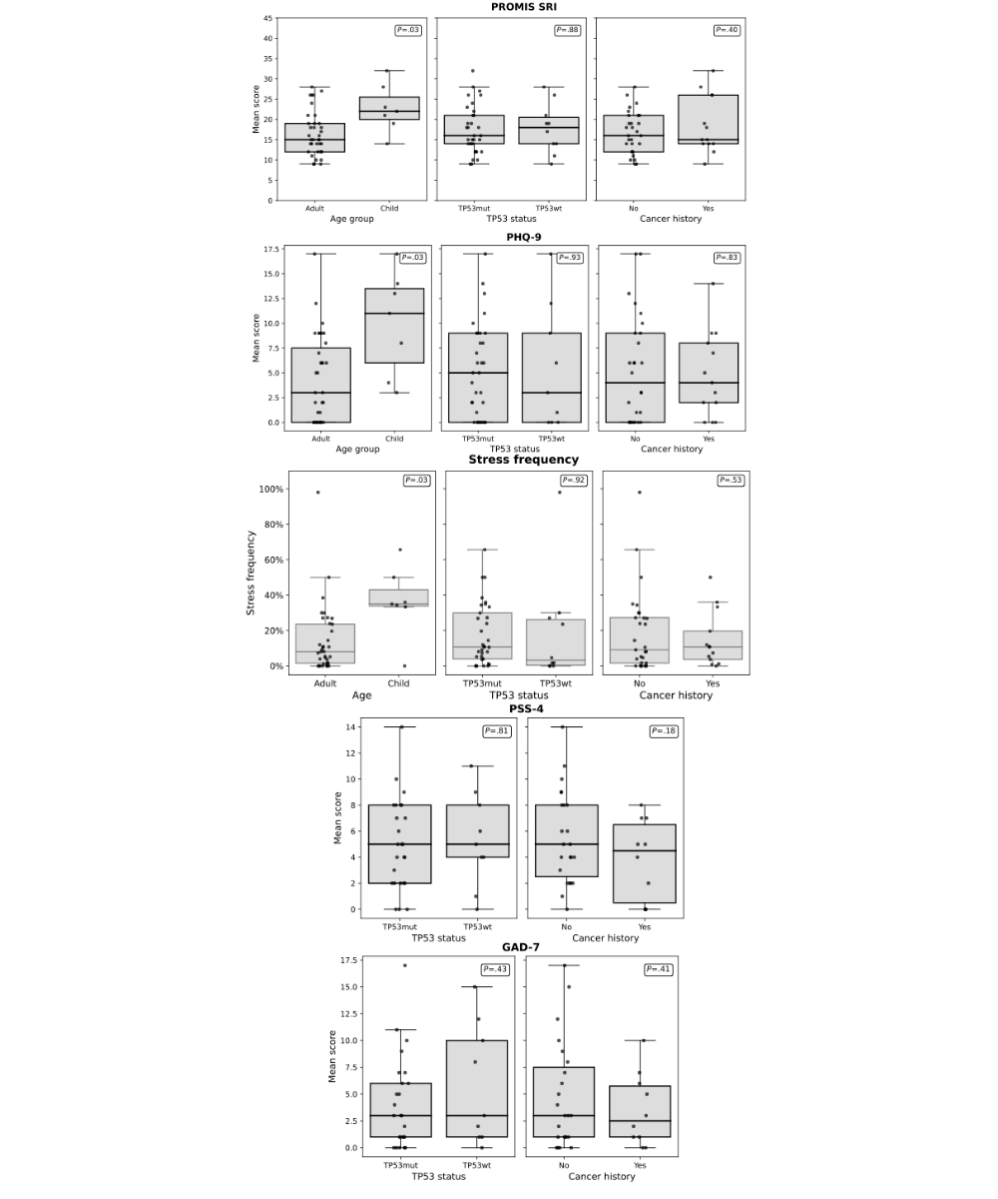
Distributions of baseline survey scores by group, plotted one survey per row. From top row to bottom row: PROMIS SRI, PHQ-9, stress frequency, PSS-4, and GAD-7 scores are each shown across the 3 groupings (age group, TP53 status, and cancer history). GAD-7: Generalized Anxiety Disorder-7; PHQ-9: Patient Health Questionnaire-9; PROMIS SRI: Patient-reported Outcomes Measurement Information System Sleep-related Impairment; PSS-4: Perceived Stress Scale-4.

Figure 6 presents the distribution of these scores across all subgroups in our study. Children exhibited significantly higher mean PHQ-9 (mean 10.0*,* SD 5.2 vs mean 4.2, SD 4.4; *t*_7_*_._*_8_=*−*2*.*8*; P*=*.*03) and PROMIS SRI (mean 22.7, SD 5.9 vs mean 16.5, SD 5.5; *t*_8_*_._*_1_=*−*2*.*58; *P*=*.*03) scores compared to adults, indicating greater levels of depressive symptoms and sleep-related impairment, respectively (Figure 6). Children also reported being “very stressed” significantly more often than adults (mean 36.3%, SD 19.9% vs mean 14.3%, SD 19.2; *t*_8_*_._*_3_=*−*2*.*7; *P*=*.*03); Figure 6). Interestingly, we found no differences in overall stress reporting, anxiety (GAD-7), or depression (PHQ-9) when stratifying by previous cancer history or *TP53* mutation status.

We use the heat map in Figure S4 in Multimedia Appendix 1 to provide a dynamic visualization of the rolling mean scores for GAD-7, PHQ-9, PROMIS SRI, PSS-4, and daily stress indicators across different participants over the study period. This visualization allows us to observe how patients transition in and out of periods of high stress, depression, and anxiety. Notably, the heat map reveals fluctuations in psychological and emotional states between clinical visits that may otherwise go unnoticed if these are not recorded. The markers indicating scan events may help to correlate these transitions with specific clinical activities, providing insights into how such events may impact patient well-being.

### Flagging Critical Mental Health Events

The PHQ-9 survey asks the respondent about self-harm and suicide-related thoughts. If a participant responded positively to this question via the app, a built-in alert mechanism was activated, immediately notifying the principal investigators of the study who were the treating clinicians for some participants. This alert system was used to assess risk for and prevent future suicide-related behaviors.

In the course of our study, this alert system was triggered by 5 (11%) participants. Notably, 3 participants belonged to the same family, comprising 2 adolescent siblings and their mother. This unique cluster of participants, all encountering similar psychological distress, presented a compelling focal point for further investigation into the triggers of such emotional distress, which, in this context, were predominantly linked to their experiences with LFS.

All participants who triggered the alert were swiftly and comprehensively assessed by a dedicated clinician, ensuring that their emotional well-being was attended to with urgency and care. This response underscores the importance of monitoring the well-being of individuals and families grappling with the complexities of LFS and the emotional challenges it presents.

### Sensor-Based Measures to Examine “Scanxiety”

During the study, 17 unique participants underwent clinical surveillance visits, which included medical imaging (eg, ultrasound or MRI) to screen for malignancy. These visits are typically preceded by stress and anxiety due to potential diagnoses. While the survey and app provided psychosocial insights, we investigated physiological signatures from the smartwatch coinciding with these surveillance events, observing notable inter- and intrapatient variability.

**Figure 7:**
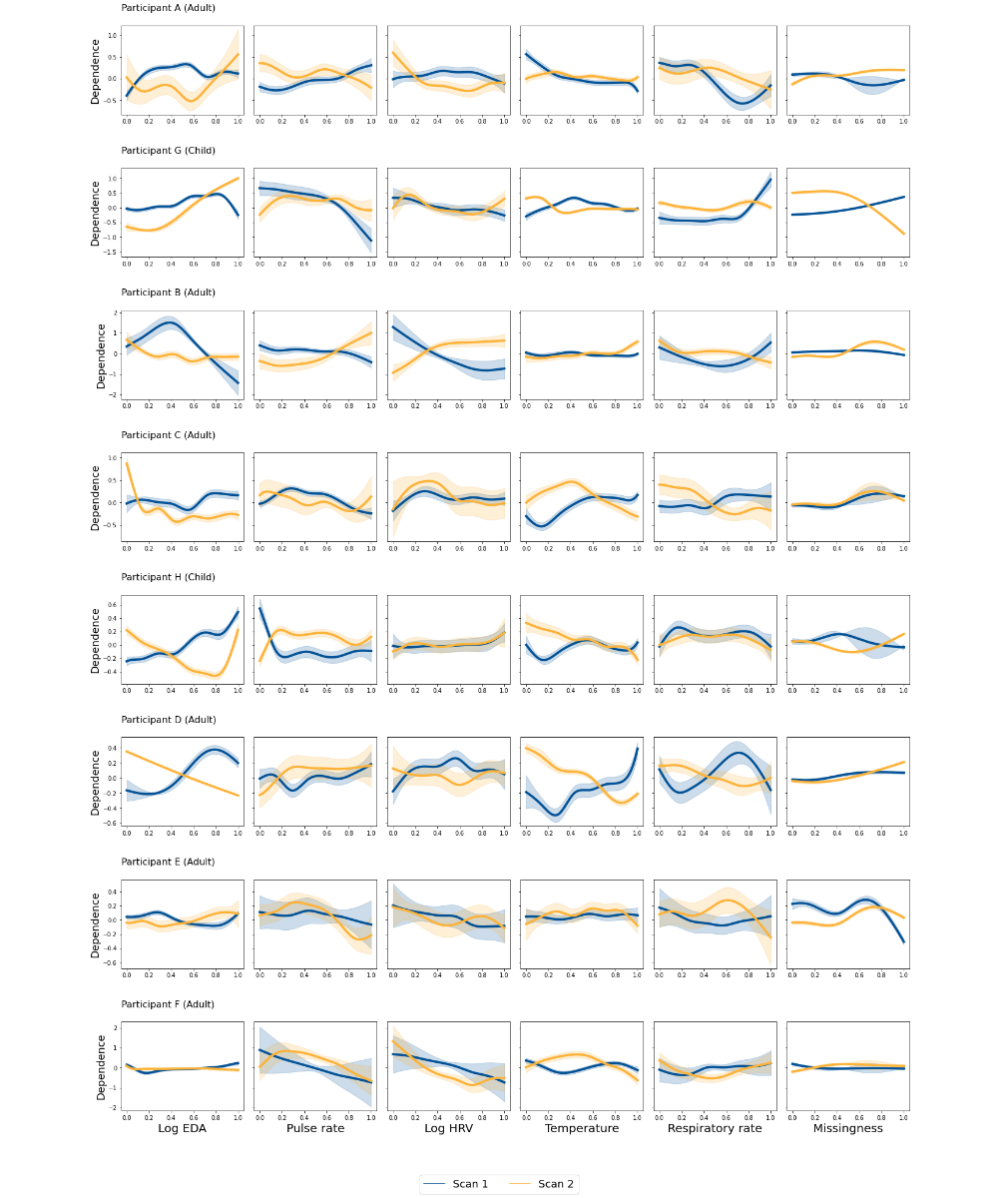
Partial dependence plots for various physiological features from a generalized additive model show individual participants (each row) with more than one scan. Overlaid plots for each scan are displayed in blue (scan 1) and yellow (scan 2). EDA: electrodermal activity; HRV: heart rate variability.

As described in the “Methods” section, 9 participants with at least 2 surveillance visits were included in this analysis. No interaction terms were included in the GAMs to balance interpretability with variance explained. Partial dependence plots (Figure 7) demonstrated significant interindividual heterogeneity, with unique patterns of dependence for each physiological measure and proximity to scans. This underscores the individualized nature of physiological responses to clinical surveillance. Of note is the similarity in dependence pattern within the same individual across different scans—indicating that some relationship between physiological feature and time-to-scan is conserved within individuals.

### Patient Perspectives and Unforeseen Consequences

**Figure 8:**
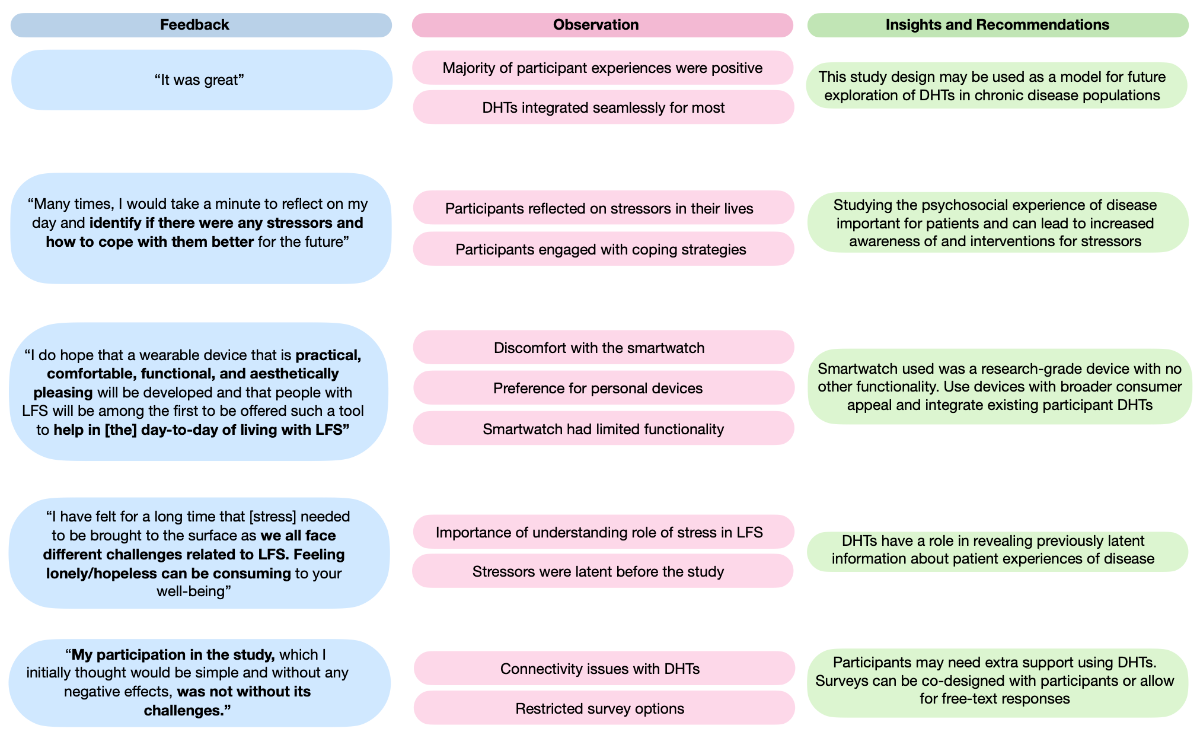
Qualitative feedback was elicited from study participants through an end-of-study questionnaire and interview. DHT: Digital Health Tool; LFS: Li-Fraumeni syndrome.

Qualitative feedback was elicited from study participants through an end-of-study questionnaire and interview (Figure 8). Participants described their experiences during the research and offered insights into the advantages and drawbacks of using smartwatches for stress assessment in the context of LFS.

In sum, the detailed feedback from the study underscores the complex emotional state of individuals with LFS and the role of integrated psychosocial support systems. The findings highlight the value of DHTs in monitoring stress and the pivotal role of safety protocols like the survey alert system in providing immediate care to those in acute distress.

Summarized feedback (ie, key quotations) highlighted various observations about study strengths and weaknesses which can inform insights and recommendations for future work using DHTs.

## Discussion

### Principal Findings

Our findings underscore the implications of integrating DHTs into clinical care for children and families with LFS. While DHTs offered valuable insights into patients’ psychological and emotional states, maintaining engagement was particularly challenging for children and adolescents. Physiological data from DHTs identified changes linked to surveillance visits, demonstrating the potential to model iatrogenic effects like “scanxiety”—stress and anxiety triggered by clinical monitoring. Qualitative feedback showed general enthusiasm for the study but pointed out areas for enhancing engagement and smartwatch usability in future DHT research. This study serves as a benchmark for larger investigations and informs the design of DHT-based interventions in chronic disease. Although our findings may extend to other chronic conditions, further work is needed for external validation.

With respect to our first objective, engagement with DHTs varied by *TP53* status, cancer history, and age, with overall survey completion exceeding 50% and higher smartwatch engagement among adults. Using multiple DHTs (eg, surveys and smartwatches) appeared to support engagement, as indicated by the strong correlation in intermodality usage among the population [26,27]. Engagement also showed cyclical patterns, likely influenced by biweekly check-ins from study organizers, consistent with trends reported in other studies [8,28]. Despite a median (IQR) smartwatch retention time of 153 (119-179) days in adults and 77 (36-151) days in children, overall engagement declined over time. Participant feedback highlighted challenges related to device practicality, comfort, and aesthetics, emphasizing the need for seamless integration of DHTs into daily life to sustain engagement. Future studies should address these barriers to facilitate longer-term observation.

Our second objective was to assess patient perspectives, as LFS imposes significant psychological and emotional burdens [29,30]. In this population, we found increased stress, higher PHQ-9 scores (depression), and sleep impairment among children compared to adults. These findings align with prior research [17,31], reinforcing the need for mental health interventions in those with chronic illness requiring surveillance. Notably, self-harm alerts were triggered through app-based PHQ-9 surveys, highlighting the utility of DHTs in facilitating timely interventions that might otherwise go unnoticed. Our qualitative analysis revealed that DHT integration encouraged patients to reflect on their existing stressors, and some conveyed optimism about the potential for future tools to better identify or reduce stress.

Our final objective was to explore the benefits for clinicians. Traditional care models provide limited snapshots of patient well-being during visits [32]. DHTs offer continuous psychosocial and physiological data between appointments, revealing insights into patient experiences that would otherwise remain latent. GAM analysis demonstrated inter- and intraindividual heterogeneity in physiological responses to clinical visits, highlighting the potential of wearable sensors to detect iatrogenic effects like “scanxiety.” Future research should explore the physiological signatures of clinical surveillance to develop early warning systems and proactive interventions. Integrating DHT data from surveys and smartwatches into clinician dashboards could enable real-time monitoring of patient well-being during treatment and follow-up.

Study limitations include a small sample size and a niche patient population, which may affect generalizability. In particular, this is a patient population with extensive clinical follow-up built into their existing care model—engagement may be reduced in populations where touchpoints to care are sparse. Our diverse population of children and adults identified unique age-specific differences: specifically, that the burden of stress is higher in children, but engagement is worse. Future studies should aim to better understand this gap, as the subgroup that may benefit the most from DHTs is also the most difficult to engage. Unlike many prior studies focused exclusively on individuals with active disease, our cohort included unaffected family members who also participate in lifelong disease surveillance, highlighting both the potential for monitoring caregivers and the broader burden of “scanxiety” experienced across entire families rather than individual patients. That being said, families with LFS are not unique to the phenomenon of “scanxiety”—surveillance is common practice in oncological [33,34] as well as nononcological disease [35]. Our insights about DHT integration may generalize to other populations monitored for chronic disease as well. Other limitations may have affected our findings as well; participants reported issues with smartwatch comfort and functionality, as the research-grade Empatica smartwatch lacked consumer features such as messaging or apps. Some felt the smartwatch was a constant reminder of their disease, potentially reducing engagement. This highlights the importance of selecting DHTs that seamlessly integrate into patients’ daily lives. Technical challenges, such as missing data and imputation, may have introduced bias into our GAM analysis, though we mitigated these effects by explicitly modeling missingness and analyzing only individuals with sufficient data.

### Conclusion

In conclusion, this study demonstrated the use of wearable technology and smartphone app surveys to engage children and families affected by LFS. The diverse dataset, spanning various ages, families, and *TP53* mutation statuses, has been made publicly available to facilitate further research and advancements in understanding and managing LFS. Participant feedback provided valuable insights for improving future digital health studies, particularly at larger scales. While DHTs hold significant promise in chronic disease management and psychosocial monitoring, larger studies are needed to optimize their application and address potential challenges.

## Appendix

Multimedia Appendix 1 Additional materials including further descriptions of methods and additional figures.

## Abbreviations

DHT: digital health tool
EDA: electrodermal activity
FFT: fast fourier transform
GAD-7: generalized anxiety disorder-7
GAM: generalized additive model
HPC: high-performance computing
HRV: heart rate variability
LFS: Li-Fraumeni syndrome
MICE: multivariate imputation via chained equations
MRI: magnetic resonance imaging
mut: mutant
PHQ-9: patient health questionnaire-9
PROMIS SRI: patient-reported outcomes measurement information system sleep-related impairment
PSS-4: perceived stress scale-4
REB: research ethics board
REDCap: research electronic data capture
wt: wild-type

## Data Availability

The datasets generated or analyzed during this study will be available open access on Sage Bionetworks.
