# Supplementary material for: Digital Health Tools Embedded in a Cancer Genetics Clinic: Observational Study
Source: JMIR Form Res. 2026 Feb 2;10:e74375. doi: 10.2196/74375 (PMC12910266; doi:10.2196/74375)
Supplement: Multimedia Appendix 1 [file formative_v10i1e74375_app1.docx]

**Supplementary Materials**

## Methods

### Data & Modeling Assumptions

We assume that observations are independent within and between groups, and that the data in each group are approximately normally distributed. We use Welch’s t-test and do not assume equal variances when comparing the means of different groups. Our survival analysis assumes that censoring is independent of survival, groups are independent, and event times are measured accurately. GAMs assume that the response variable follows an exponential family distribution (i.e., Gaussian), and that the predictors contribute additively through smooth, nonlinear functions (i.e., splines). The smoothness of each function is penalized to avoid overfitting, and multicollinearity among predictors is minimized. We note that our data consists of repeated time series measurements from wearable sensors, and thus, adjacent samples may exhibit autocorrelation. While the GAMs were fit assuming independence of observations, we mitigated this concern partially by normalizing within participants and using penalized splines with cross-validation. Future work may benefit from explicitly modeling temporal structure (e.g., autoregressive terms or mixed-effect GAMs).

These are all standard assumptions frequently used in this type of analysis.


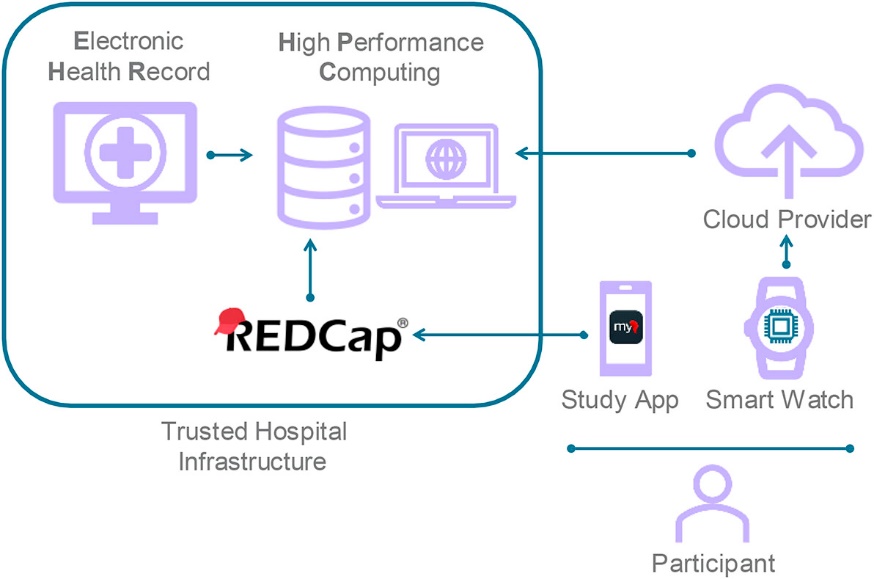


**Figure S1.** Schematic of the data integration framework. Participants used a smartwatch and study app to collect physiological and survey data. Data from the smartwatch is sent to a cloud provider, while survey data is stored in REDCap. Both data sources, along with electronic health record data, are transferred to a high-performance computing environment within a trusted hospital infrastructure for integration and analysis.


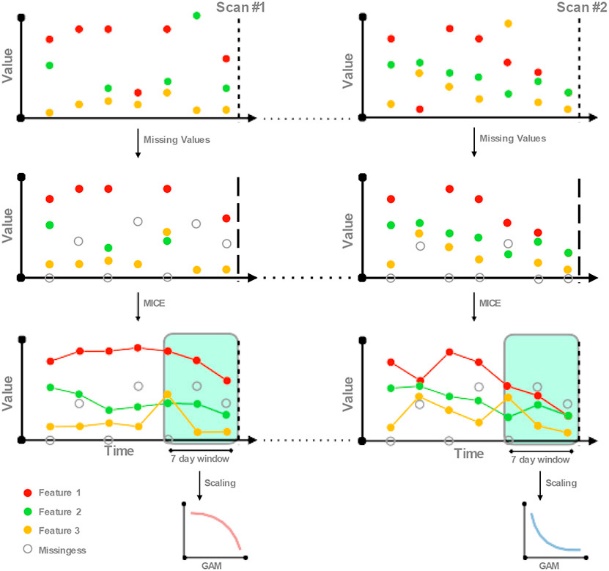


**Figure S2.** Schematic of data preprocessing for GAM analysis. This figure shows an abstraction of sensor data from one example participant with two unique scans across the study period. After identification of missing points and MICE imputation, we fit a separate GAM for each scan using the preceding three days of smartwatch features to predict time-to-scan. For visual clarity, only three features are shown.


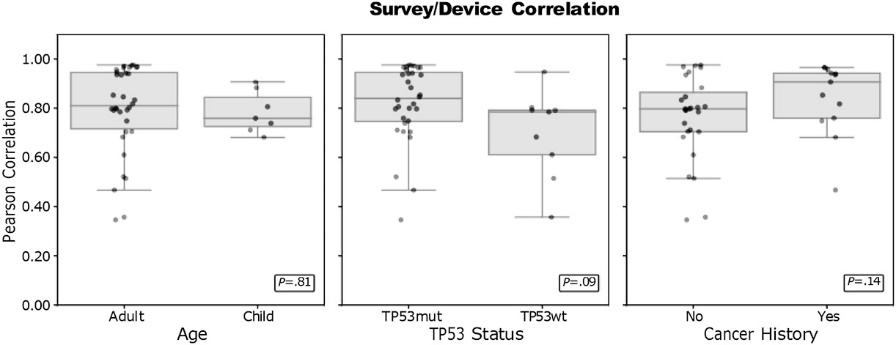


**Figure S3.** Pearson correlation coefficients between smartwatch usage and survey completion, stratified by age group (left) *TP53* status (middle) and cancer history (right). Higher correlations indicate stronger consistency between smartwatch and survey data. Mean correlation within each subgroup was generally above 0.5, suggesting good engagement and data consistency, with no statistically significant differences between subgroups.


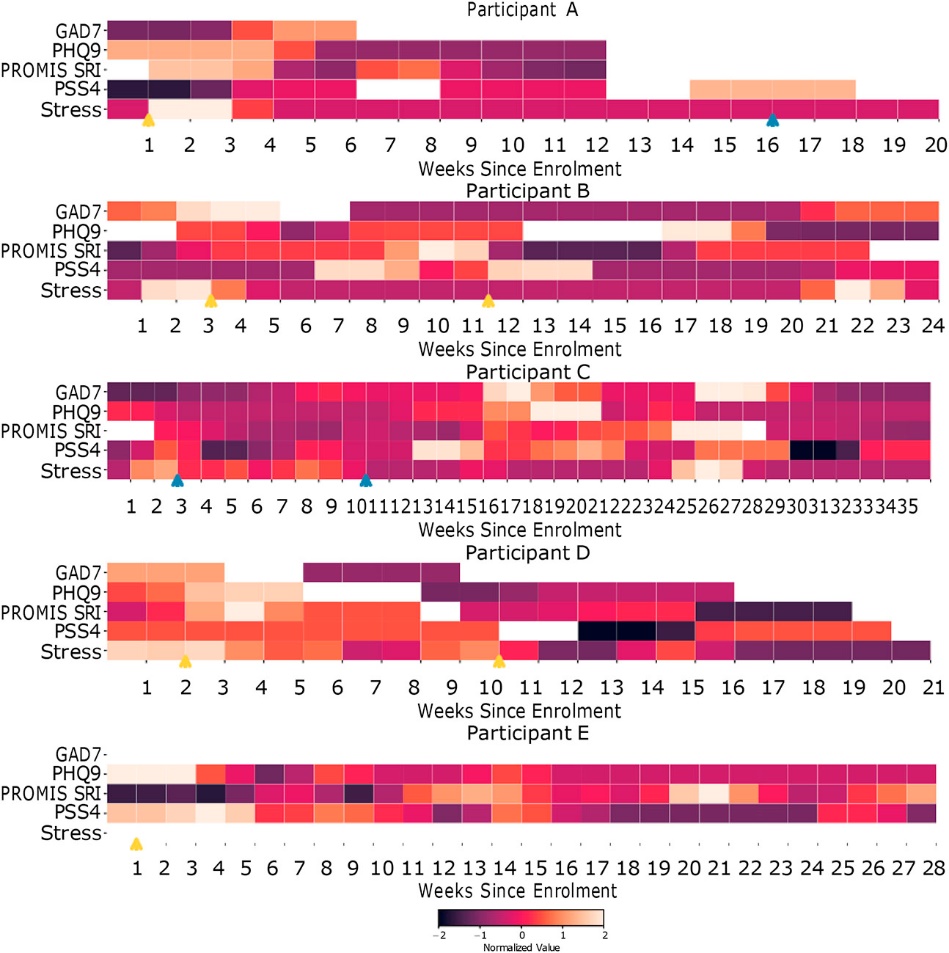


**Figure S4.** Heat map showing the rolling scores of different participants over time for GAD-7, PHQ-9, PROMIS SRI, PSS-4, and Stress measures. Red indicates more severe symptoms. Each row represents an individual participant, with weeks since enrollment displayed on the x-axis. The color gradient

highlights changes in symptom severity across the study period. **Blue Arrow** indicates a scan event for the particular participant. **Yellow Arrow** indicates their family member’s scan.

### Survey Measures

The study app (MyCap) was used only by participants aged 11 years and older, with two versions administered: an adult version for participants aged 18 years and above, and an adolescent version for those aged 11-17 years. For younger children aged 5-10 years, parents completed additional in-app parent-reported measures on their behalf. The survey selection process aimed to balance breadth of information with minimization of survey burden. We selected measures consistent with those used in previous related studies [1]. Selected surveys have been previously validated for assessing their intended constructs in relevant populations. A detailed compilation of survey measures used, frequency of administration, and completion frequency in each age group can be found in Table S1, Table S2, and Table S3.

**Table S1.** Surveys administered for participants 18+ (*n* = 36). Frequency, %completion, and description with original citations and/or validation studies shown.

| **Survey Measure** | **Frequency** | **% Completed** | **Description** |
| --- | --- | --- | --- |
| Demographics | Once | 75% | Study intake demographics captured  at study start time. |
| Daily Stress | Daily | 64% *±* 22% | Binary daily stress measure (i.e., “were  you stressed or very stressed today?”). Administered daily at the same time  as SAM survey below [1]. |
| SAM | Daily | 64% *±* 22% | Daily self-assessment measures (SAM)  Scales: Rates mood, stress, energy, cog-  nition on 5-point icon scale [1]. |
| PROMIS – PI | Weekly | 69% *±* 22% | PROMIS Pain Interference: 6-question  survey assessing the consequences of  pain on life activities[1,2]. |
| PROMIS – SRI | Weekly | 71% *±* 21% | PROMIS Sleep-Related Impairment:  5-question survey assessing sleep-related alertness, tiredness, and day-  time function [1,3]. |
| PSS-4 | Biweekly | 77% *±* 16% | Perceived Stress Scale - 4: A 4-item  measure of perceived stress [4]. |
| FAS | Biweekly | 77% *±* 17% | Fatigue Assessment Scale: 10-question  survey assessing fatigue severity [5]. |
| PHQ-9 | Biweekly | 73% *±* 22% | PHQ-9: 9-question survey assessing  depressive symptoms over the past 2  weeks [6]. |
| GAD7 | Biweekly | 74% *±* 22% | GAD-7: 8-question survey assessing  anxiety over the past 2 weeks [7]. |
| PROMIS – Global | Monthly | 87% *±* 18% | PROMIS Global-10: 10-question sur-  vey assessing overall physical health, mental health, social health, pain,  fatigue, and quality of life [8]. |
| PROMIS – ES | Monthly | 86% *±* 17% | PROMIS Emotional Support 4a: 4-  question survey assessing available  emotional support [9]. |

**Table S2.** Surveys administered for participants 11-17 (*n* = 7). Frequency, % completion, and description with original citations and/or validation studies shown.

| **Survey Measure** | **Frequency** | **% Completed** | **Description** |
| --- | --- | --- | --- |
| Demographics | Once | 75% | Study intake demographics captured  at study start time. |
| Daily Stress | Daily | 51% *±* 22% | Binary daily stress measure (i.e., “were  you stressed or very stressed today?”). Administered daily at the same time  as SAM survey below. |
| SAM | Daily | 51% *±* 22% | Daily self-assessment measures (SAM)  Scales: Rates mood, stress, energy, cog-  nition on 5-point icon scale. |
| PROMIS – PI | Weekly | 59% *±* 24% | Patient-Reported Outcomes Measurement Information System (PROMIS) Pediatric Pain Interference - Short Form 8a: 8-item survey assessing self- reported consequences of pain on life  activities [10]. |
| PROMIS – SRI | Weekly | 79% *±* 28% | PROMIS Sleep-Related Impairment  (pediatric version): 5-question survey assessing sleep-related alertness, tired-  ness, and daytime function [11]. |
| FAS | Biweekly | 84% *±* 18% | Fatigue Assessment Scale: 10-question  survey assessing fatigue severity. |
| PHQ-9A | Biweekly | 79% *±* 25% | PHQ-9A: 12-item measure of depression adapted from PHQ-9 for adolescents [12]. |
| CSQ-CA | Biweekly | 69% *±* 20% | Chronic Stress Questionnaire for Children and Adolescents: 17-item self-report measure of chronic stress in  children and adolescents [13]. |
| PROMIS-PA | Biweekly | 87% *±* 22% | PROMIS Pediatric Anxiety - Short  Form 8a: 8-item measure of generalized  anxiety in children and teens [14]. |
| PROMIS-PFR | Monthly | 90% *±* 21% | PROMIS Pediatric Family Relation-  ships - Short Form 4a: 4-question survey about children’s feelings toward  family relationships [15]. |
| PedsQL | Monthly | 96% *±* 8% | Pediatric Quality of Life (PedsQL)  - Cancer Module: 26-item self-report and parent-report measure of quality  of life [16]. |

**Table S3.** Surveys administered for participants 5-10 (*n* = 2). Frequency, % completion, and description with original citations and/or validation studies shown.

| **Survey Measure** | **Frequency** | **% Completed** | **Description** |
| --- | --- | --- | --- |
| PROMIS – PI | Weekly | 82% *±* 0% | PROMIS Parent Proxy Pain Interference - Short Form 8a: 8-item parent- reported survey assessing a child’s  pain-related impacts on daily life. |
| PROMIS – SRI | Weekly | 82% *±* 0% | PROMIS Sleep-Related Impairment  (pediatric version): 5-question survey assessing sleep-related alertness, tired-  ness, and daytime function. |
| MFQ | Biweekly | 75% *±* 0% | Mood and Feelings Questionnaire -  Short Version: 13-item parent-reported measure on a child’s feelings and  behaviors over the past two weeks [17]. |
| PedsQL – MFS | Biweekly | 79% *±* 0% | PedsQL Multidimensional Fatigue  Scale: 18-item parent-reported survey exploring fatigue severity in children. |
| SCASP-8 | Biweekly | 64% *±* 0% | Spence Children’s Anxiety Scale - Parent Report - Brief Version (SCASP-8): Parent-reported measure of generalized anxiety [18]. |
| CSQ-CA | Monthly | 71% *±* 0% | Chronic Stress Questionnaire for Children and Adolescents: 17-item self- reported measure of chronic stress in  children and adolescents. |

**Table S4.** Dataset characteristics and model performance for GAM analyses. For each participant, *n* is the number of observations and *d* = 6 is the feature dimension. % MSE indicates the 5-fold cross-validation mean-squared error *±* standard deviation.

| Participant, scan number | (*n, d*) | % MSE *±* St.Dev |
| --- | --- | --- |
| A, scan 1 | (10080, 6) | 6.53 *±* 0.20 |
| A, scan 2 | (10080, 6) | 7.08 *±* 0.14 |
| B, scan 1 | (10080, 6) | 6.93 *±* 0.59 |
| B, scan 2 | ( 8640, 6) | 7.58 *±* 0.14 |
| C, scan1 | (10080, 6) | 5.29 *±* 0.23 |
| C, scan 2 | (10080, 6) | 6.44 *±* 0.10 |
| D, scan 1 | (10080, 6) | 5.84 *±* 0.15 |
| D, scan 2 | (10080, 6) | 4.24 *±* 0.13 |
| E, scan 1 | ( 1740, 6) | 2.00 *±* 0.15 |
| E, scan 2 | (10080, 6) | 7.77 *±* 0.15 |
| F, scan 1 | (10080, 6) | 7.40 *±* 0.12 |
| F, scan 2 | (10080, 6) | 7.47 *±* 0.14 |
| G, scan 1 | (10080, 6) | 3.84 *±* 0.12 |
| G, scan 2 | (10080, 6) | 4.86 *±* 0.11 |
| H, scan 1 | (10080, 6) | 6.81 *±* 0.08 |
| H, scan 2 | (10080, 6) | 5.99 *±* 0.14 |

**References**

[1] *Goodday SM, Karlin E, Alfarano A, Brooks A, Chapman C, Desille R, Rangwala S, Karlin DR, Emami H, Woods NF, Boch A, Foschini L, Wildman M, Cormack F, Taptiklis N, Pratap A, Ghassemi M, Goldenberg A, Nagaraj S, Walsh E, Stress And Recovery Participants, Friend S. An alternative to the light touch digital health remote study: the stress and recovery in frontline COVID-19 health care workers study. JMIR Form Res 2021; 5(12):e32165*

[2] *Amtmann D, Cook KF, Jensen MP, Chen WH, Choi S, Revicki D, Cella D, Rothrock N, Keefe F, Callahan L, Lai J-S. Development of a PROMIS item bank to measure pain interference. Pain 2010; 150(1):173-182*

[3] *Buysse D, Yu L, Moul DE, Germain A, Stover A, Dodds NE, Johnston KL, Shablesky-Cade MA, Pilkonis PA. Development and validation of patient-reported outcome measures for sleep disturbance and sleep-related impairments. Sleep 2010; 33(6):781-792*

[4] *Cohen S, Kamarck T, Mermelstein R. A global measure of perceived stress. J Health Soc Behav 1983; 24(4):385-396*

[5] *Michielsen HJ, De Vries J, Van Heck GL. Psychometric qualities of a brief self-rated fatigue measure: The Fatigue Assessment Scale. J Psychosom Res 2003; 54(4):345-352*

[6] *Kroenke K, Spitzer RL, Williams JBW. The PHQ-9: validity of a brief depression severity measure. J Gen Intern Med 2001; 16(9):606-613*

[7] *Spitzer RL, Kroenke K, Williams JBW, Löwe B. A brief measure for assessing generalized anxiety disorder: the GAD-7. Arch Intern Med 2006; 166(10):1092-1097*

[8] *Hays RD, Bjorner JB, Revicki DA, Spritzer KL, Cella D. Development of physical and mental health summary scores from the patient-reported outcomes measurement information system (PROMIS) global items. Qual Life Res 2009; 18(7):873-880*

[9] *Hahn EA, DeWalt DA, Bode RK, Garcia SF, DeVellis RF, Correia H, Cella D, PROMIS Cooperative Group. New English and Spanish social health measures will facilitate evaluating health determinants. Health Psychol 2014; 33(5):490-499*

[10] *Varni JW, Stucky BD, Thissen D, Dewitt EM, Irwin DE, Lai J, Yeatts K, Dewalt D A. PROMIS pediatric pain interference scale: an item response theory analysis of the pediatric pain item bank. J Pain 2010; 11(11):1109-1119*

[11] *Bevans KB, Meltzer LJ, De La Motte A, Kratchman A, Viél D, Forrest CB. Qualitative development and content validation of the PROMIS pediatric sleep health items. Behav Sleep Med 2019; 17(5):657-671*

[12] *Richardson L, McCauley E, Grossman DC, McCarty CA, Richards J, Russo JE, Rockhill C, Katon W. Evaluation of the Patient Health Questionnaire-9 item for detecting major depression among adolescents. Pediatrics 2010; 126(6):1117-1123*

[13] *de Bruin EI, Sieh DS, Zijlstra BJH, Meijer A. Chronic childhood stress: psychometric properties of the chronic stress questionnaire for children and adolescents (CSQ-CA) in three independent samples. Child Ind Res 2017; 11(4):1389-1406*

[14] *Quinn H, Thissen D, Liu Y, Magnus B, Lai J, Amtmann D, Varni JW, Gross HE, DeWalt DA. Using item response theory to enrich and expand the PROMIS® pediatric self report banks. Health Qual Life Outcomes 2014; 12:160*

[15] *Bevans KB, Riley AW, Landgraf JM, Carle AC, Teneralli RE, Fiese BH, Meltzer LJ, Ettinger AK, Becker BD, Forrest CB. Children's family experiences: development of the PROMIS pediatric family relationships measures. Qual Life Res 2017; 26(11):3011-3023*

[16] *Varni JW, Seid M, Rode CA. The PedsQL: measurement model for the pediatric quality of life inventory. Med Care 1999; 37(2):126-139*

[17] *Thabrew H, Stasiak K, Bavin L, Frampton C, Merry S. Validation of the mood and feelings questionnaire (MFQ) and short mood and feelings questionnaire (SMFQ) in New Zealand help-seeking adolescents. Int J Methods Psychiatr Res 2018; 27(3):e1610*

[18] *Reardon T, Spence SH, Hesse J, Shakir A, Creswell C. Identifying children with anxiety disorders using brief versions of the spence children's anxiety scale for children, parents, and teachers. Psychol Assess 2018; 30(10):1342-1355*
